# Supplementary material for: Distinct functional domains of Dystroglycan regulate inhibitory synapse formation and maintenance in cerebellar Purkinje cells
Source: bioRxiv. 2024 Aug 30:2024.08.29.610348. Preprint. [Version 1] doi: 10.1101/2024.08.29.610348 (PMC11383678; doi:10.1101/2024.08.29.610348)
Supplement: Supplement 1 [file NIHPP2024.08.29.610348v1-supplement-1.pdf]

bioRxiv preprint doi: https://doi.org/10.1101/2024.08.29.610348; this version posted August 30, 2024. The copyright holder for this preprint (which was not certified by peer review) is the author/funder. This article is a US Government work. It is not subject to copyright under 17 USC 105 and is also made available for use under a CC0 license.

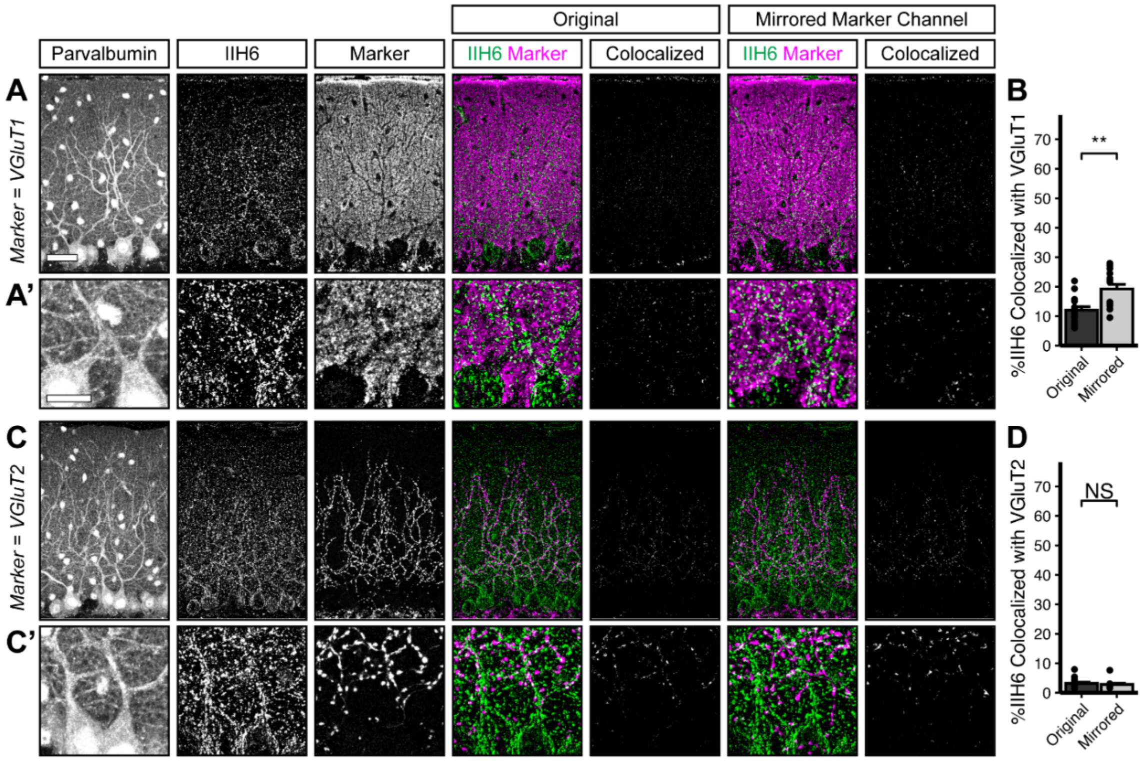

Supplemental Figure 1. Dystroglycan does not co-localize with markers of excitatory synapses.

Cerebellar cortex of lobules V-VI were immunostained with Parvalbumin to show Purkinje cell and MLI morphology and counterstained with IIH6 (glycosylated Dystroglycan) and VGluT1 (parallel fibers) (**A**) or VGluT2 (climbing fibers) (**C**). Both the merged channels (IIH6, green; VGluT1/VGluT2, magenta) and colocalized pixels are shown for the original image and for original IIH6 with the mirrored VGluT1/VGluT2 channel. Images are maximum projections. (**B, D**) Quantification of the percent of IIH6 puncta that are colocalized with VGluT1/VGluT2 puncta. Scale bar for (**A**, **C**) is 50µm; scale bar for insets (**A'**, **C'**) is 25µm. VGluT1 N = 15 images, 3 animals. VGluT2 N = 15 images, 3 animals.

bioRxiv preprint doi: https://doi.org/10.1101/2024.08.29.610348; this version posted August 30, 2024. The copyright holder for this preprint (which was not certified by peer review) is the author/funder. This article is a US Government work. It is not subject to copyright under 17 USC 105 and is also made available for use under a CC0 license.

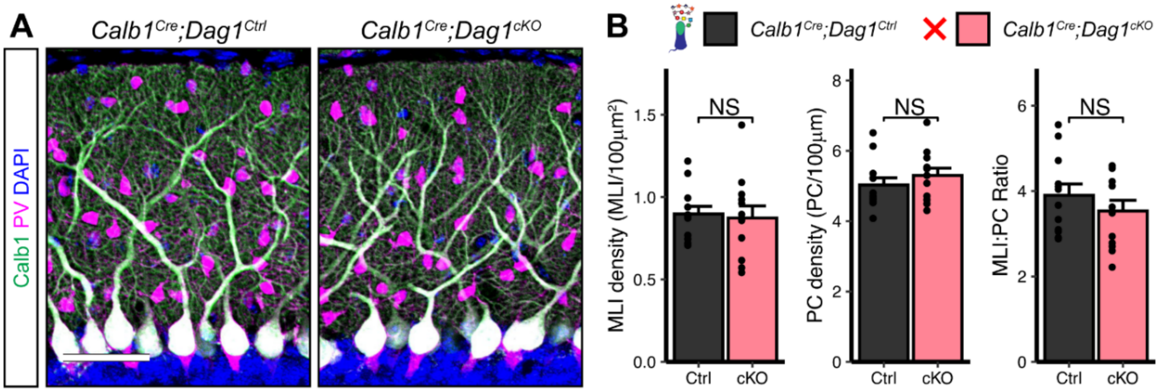

Supplemental Figure 2. Purkinje and MLI cell counts are unchanged in *Calb1^{Cre};Dag1^{cKOs}*.

(A) *Calb1^{Cre};Dag1^{cKO}* and littermate controls immunostained for Calbindin (Purkinje cells, green) and Parvalbumin (Purkinje cells and MLIs, magenta). Nuclei are shown in blue. Images are maximum projections. Scale bar = 50µm. **(B)** Quantification of MLI density, Purkinje cell density, and the ratio of MLIs to Purkinje cells. Error bars represent mean + SEM. *Calb1^{Cre};Dag1^{Ctrl}* N = 12 ROIs, 6 images, 3 animals. *Calb1^{Cre};Dag1^{cKO}* N = 12 ROIs, 6 images, 3 animals.

bioRxiv preprint doi: https://doi.org/10.1101/2024.08.29.610348; this version posted August 30, 2024. The copyright holder for this preprint (which was not certified by peer review) is the author/funder. This article is a US Government work. It is not subject to copyright under 17 USC 105 and is also made available for use under a CC0 license.

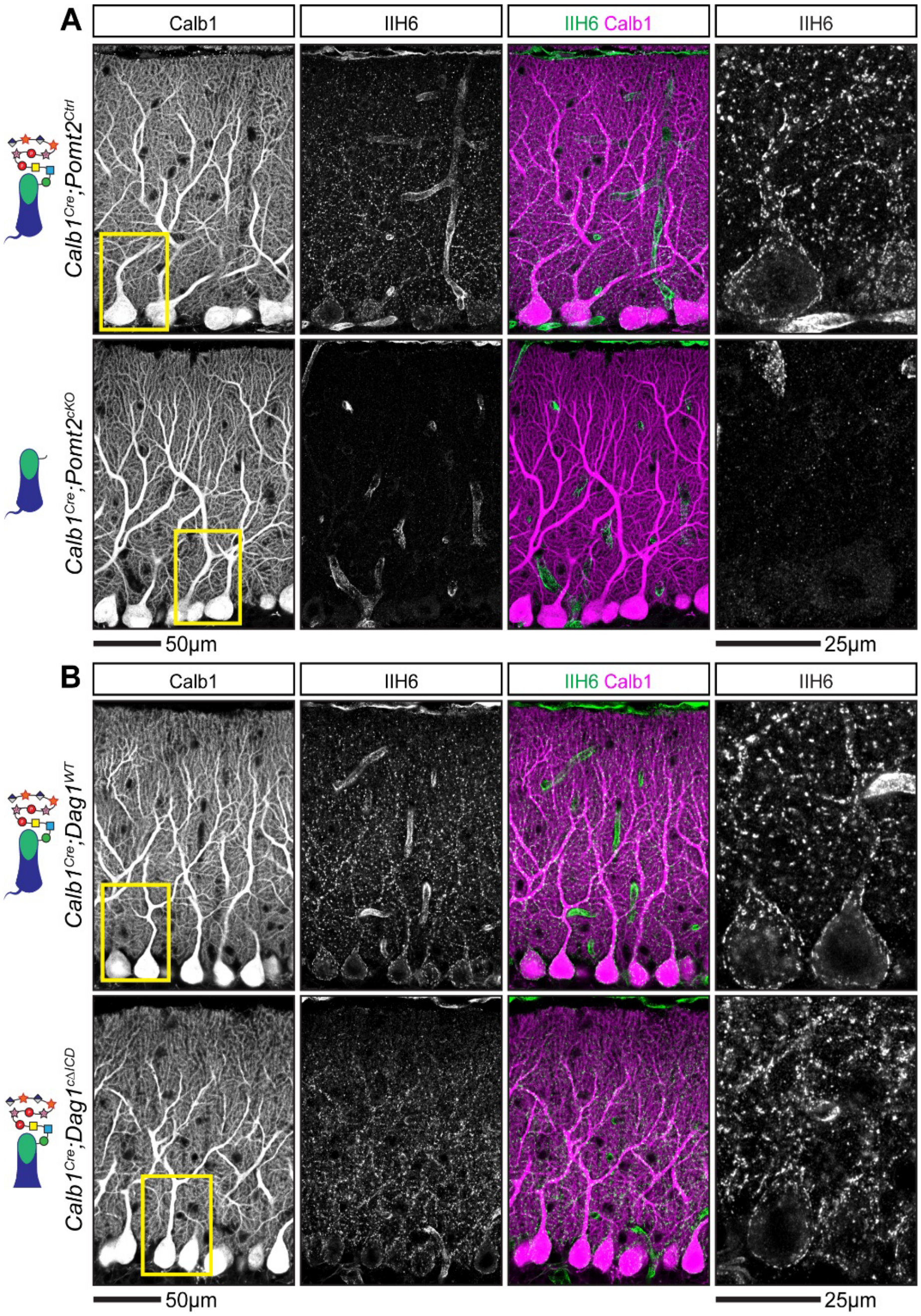

Supplemental Figure 3. Localization of matriglycan chains on Dystroglycan in *Calb1^{Cre};Pomt2^{cKO}* and *Calb1^{Cre};Dag1$^{c\Delta ICD}$* Purkinje cells.

(A-B) Cerebellar sections from *Calb1^{Cre};Pomt2^{cKOs}* and littermate controls **(A)** or *Calb1^{Cre};Dag1$^{c\Delta ICDs}$* and littermate controls **(B)** were immunostained for Calbindin (magenta), to visualize Purkinje cells, along with IIH6 (green), to visualize matriglycan chains on Dystroglycan. The rightmost panel represents a magnified view of the area outlined in yellow in the leftmost low magnification panel. Images are maximum projections.
